# Evaluating the Utility of Prostate-Specific Antigen Density in Risk Stratification of PI-RADS 3 Peripheral Zone Lesions on Non-Contrast-Enhanced Prostate MRI: An Exploratory Single-Institution Study

**DOI:** 10.7759/cureus.41369

**Published:** 2023-07-04

**Authors:** Emilian Kalchev

**Affiliations:** 1 Diagnostic Imaging, St Marina University Hospital, Varna, BGR

**Keywords:** prostate cancer, peripheral zone, multi-parametric mri, pi-rads 3 lesions, prostate specific antigen density (psad)

## Abstract

Objective

This study aimed to explore the potential of prostate-specific antigen density (PSAD) as a supplementary tool for defining high-risk Prostate Imaging Reporting and Data System (PI-RADS) 3 lesions in the peripheral zone on non-contrast-enhanced MRI. This additional stratification tool could supplement the decision-making process for biopsy, potentially helping in identifying higher-risk patients more accurately, minimizing unnecessary procedures in lower-risk patients, and limiting the need for dynamic contrast-enhanced (DCE) scans.

Materials and methods

Between January 2019 and April 2023, 30 patients with PI-RADS 3 lesions underwent MRI-ultrasound fusion biopsies at our institution. Age and PSAD values were investigated using logistic regression and chi-square automatic interaction detection (CHAID) analysis to discern their predictive value for malignancy.

Results

The mean patient age was 64.7 years, and the mean PSAD was 0.13 ng/mL^2^. Logistic regression demonstrated PSAD to be a significant predictor of cancer (p=0.012), but not age (p=0.855). CHAID analysis further identified a PSAD cut-off value of 0.12, below which the cancer detection rate was 23.1% and above which the rate increased to 76.5%.

Conclusions

This exploratory study suggests that PSAD might be utilized to enhance the stratification of high-risk PI-RADS 3 lesions in the peripheral zone on non-contrast-enhanced MRI, aiding in decision-making for biopsy. While biopsy remains the gold standard for definitive diagnosis, a high PSAD value may suggest a greater need for biopsy in this specific group. Although further validation in larger cohorts is required, our findings contribute to the ongoing discourse on optimizing PI-RADS 3 lesion management. Limitations include a small sample size, the retrospective nature of the study, and the single-center setting, which may impact the generalizability of our results.

## Introduction

Prostate cancer remains the second most common cancer in men globally and a significant cause of cancer-related death [1]. The advent of multiparametric magnetic resonance imaging (mpMRI) has revolutionized the diagnosis and management of prostate cancer, assisting clinicians to visualize tumors more accurately and perform targeted biopsies [2]. The Prostate Imaging Reporting and Data System (PI-RADS) provides a structured interpretation and reporting system for prostate MRI, with the latest version being v2.1 [3].

PI-RADS 3 lesions on mpMRI represent an indeterminate risk of clinically significant prostate cancer, which complicates clinical decision-making concerning biopsy [4,5]. This situation becomes more intricate for lesions situated in the peripheral zone, detected through non-contrast-enhanced MRI (biparametric MRI). The absence of a dynamic contrast-enhanced (DCE) sequence can restrict the upgradation of these lesions to PI-RADS 4 [6]. Hence, integrating other parameters for a more accurate risk stratification becomes necessary.

One such parameter, prostate-specific antigen density (PSAD), has been identified as a promising adjunct in patients with PI-RADS 3 lesions [6]. This measure, which combines serum prostate-specific antigen (PSA) levels and prostate volume, has been incorporated into clinical decision-making guidelines, such as the National Comprehensive Cancer Network (NCCN) guidelines for the early detection of prostate cancer [7], and has also shown potential in differentiating between benign and malignant PI-RADS 3 lesions on mpMRI [3,4,6,8]. However, we aim to explore its potential in the less invasive biparametric MRI (bpMRI), which excludes dynamic contrast enhancement from the imaging protocol.

In the current exploratory study, we assess the relationship between age, PSAD, and the presence of malignancy in patients with PI-RADS 3 lesions in the peripheral zone on non-contrast-enhanced MRI. Our intent is not to bypass biopsy but to provide a framework for clinicians to decide which patients would benefit most from the procedure, given the indeterminate risk that PI-RADS 3 lesions pose. We chose to focus on prostate cancer broadly, acknowledging that there are often discrepancies between biopsy and prostatectomy Gleason scores, which can impact the differentiation between prostate cancer and clinically significant prostate cancer [9,10]. Our findings could not only contribute to the growing body of evidence but also support a more nuanced approach to biopsy decision-making. Furthermore, understanding the role of PSAD in risk stratification of PI-RADS 3 lesions could potentially minimize the need for contrast agents, reducing costs and avoiding the side effects associated with their use.

## Materials and methods

We conducted a retrospective review of de-identified patient data from our institution between January 2019 and April 2023. Our study was exploratory in nature and did not require ethics committee approval due to the retrospective and de-identified nature of the data.

Imaging technique

We performed all imaging using a Siemens Magnetom Verio 3.0 Tesla MRI machine with a body coil. The standard bpMRI protocol for each patient included axial and sagittal T2-weighted images, axial and sagittal diffusion-weighted imaging (DWI) with b values of 50, 400, and 1000 s/mm², an apparent diffusion coefficient (ADC) map, coronal T2-weighted images with fat suppression, and axial T1-weighted DIXON images.

Patients

The study involved patients who underwent a prostate MRI and were identified as having only PI-RADS 3 lesions located in the peripheral zone, as detected through non-contrast-enhanced MRI. These lesions were identified based on the PI-RADS v2.1 criteria. Two experienced radiologists evaluated the MRI images, and any discrepancies were resolved through discussion.

We excluded patients who had poor-quality MRI images, lesions classified higher than PI-RADS 3 on the same examination, or findings of acute inflammation on pathology, as this could influence PSA levels and falsely affect the estimated PSAD threshold for stratification.

Biopsy procedure

We executed all biopsies using a transrectal approach, facilitated by MRI-ultrasound fusion guidance. This method enabled targeted sampling of the area of interest, with core biopsies taken for pathological assessment.

Data analysis

We analyzed the data using SPSS Version 21 (IBM Corp., Armonk, NY). The primary goal of our analysis was to investigate the association of age and PSAD with the presence of malignancy. This analysis was intended to support biopsy decision-making by providing additional information on which patients might be at higher risk, rather than suggesting biopsy is unnecessary. We calculated PSAD by dividing the serum PSA level by the prostate volume as reported on MRI. We used univariable and multivariable logistic regression models to identify predictors of malignancy. We selected cut-off values through CHAID (chi-square automatic interaction detection) decision tree analysis.

The patients' age and PSAD served as independent variables, while the biopsy-confirmed presence of malignancy served as the dependent variable. We set statistical significance at p<0.05.

In addition to determining the cut-off value using CHAID decision tree analysis, we also performed a receiver operating characteristic (ROC) curve analysis.

## Results

We included 30 patients with PI-RADS 3 lesions in the peripheral zone in our analysis. The participants' ages ranged from 48 to 74 years. PSAD values ranged from 0.04 to 0.23, with a median value of 0.13.

**Figure 1:**
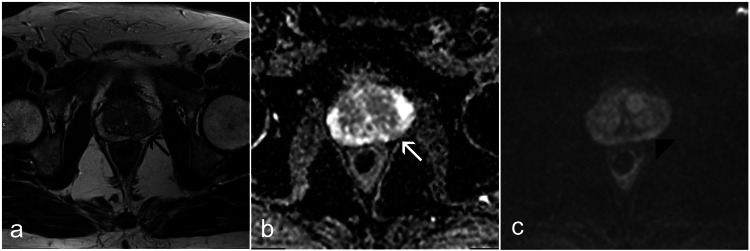
MRI of a patient with PSA density of 0.07 and benign prostatic hyperplasia on histopathology. (a) T2-weighted image showing an area of moderate hypointensity in the peripheral zone (black arrow). (b) ADC map showing a corresponding area of moderate hypointensity (white arrow). (c) High b-value (b=1000) DWI image showing mild hyperintensity within the same region (black arrowhead). This lesion was classified as PI-RADS 3 on non-contrast-enhanced MRI and confirmed to be benign on biopsy. MRI, magnetic resonance imaging; PSA, prostate-specific antigen; ADC, apparent diffusion coefficient; DWI, diffusion-weighted imaging; PI-RADS, Prostate Imaging Reporting and Data System

**Figure 2:**
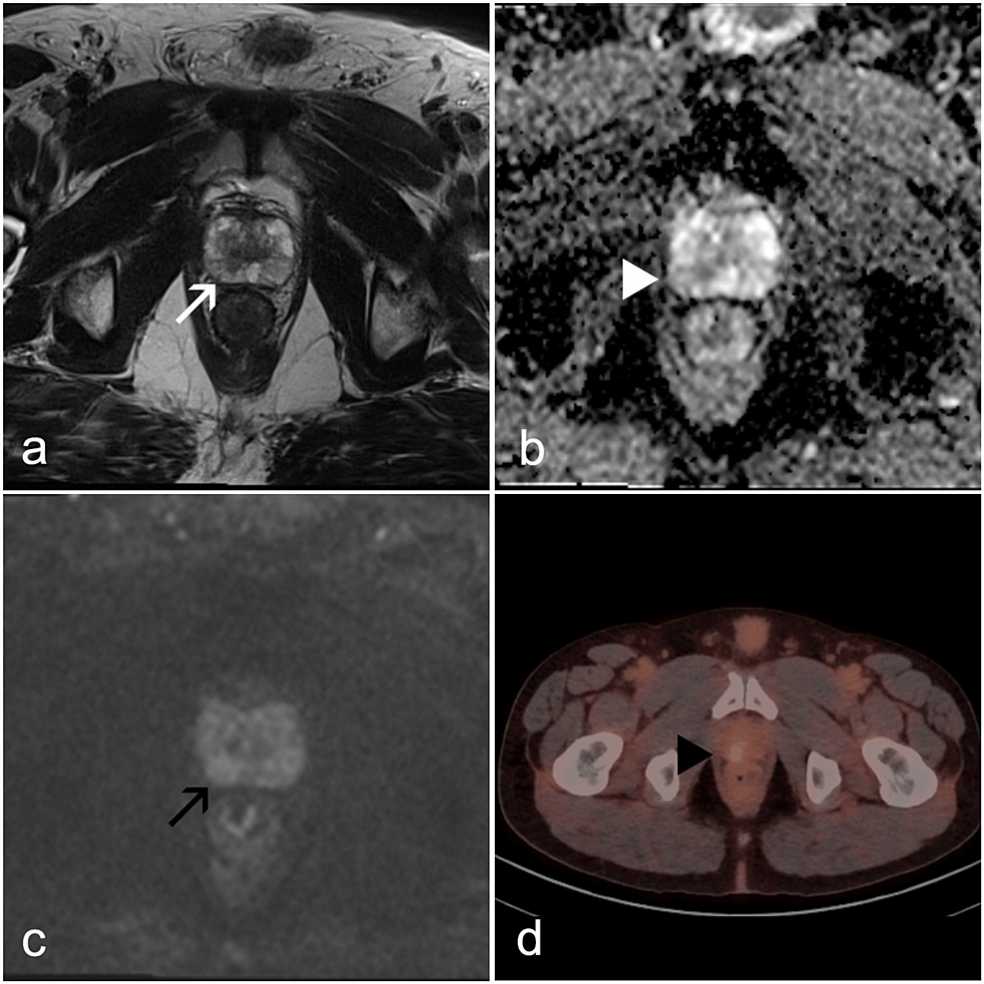
MRI and Ga-68 PSMA PET/CT of a patient with PSA density of 0.15 and histopathologically confirmed prostate adenocarcinoma (Gleason score 3+4=7). (a) T2-weighted image demonstrating a rounded area of moderate hypointensity in the peripheral zone (white arrow). (b) ADC map presenting a corresponding area of moderate hypointensity (white arrowhead). (c) High b-value (b=1000) DWI image depicting mild hyperintensity within the same region (black arrow). (d) Ga-68 PSMA PET/CT Scan showing increased metabolism at the corresponding site (black arrowhead). This lesion was classified as PI-RADS 3 on non-contrast-enhanced MRI and proven malignant on biopsy. Note: The SUVmax value is not available. MRI, magnetic resonance imaging; Ga-68 PSMA, gallium-68 prostate-specific membrane antigen; PET/CT, positron emission tomography/computed tomography; PSA, prostate-specific antigen; ADC, apparent diffusion coefficient; DWI, diffusion-weighted imaging; PI-RADS, Prostate Imaging Reporting and Data System

Multivariable logistic regression revealed that age did not significantly correlate with malignancy (p=0.855). However, PSAD displayed a significant positive association (p=0.012), suggesting that a higher PSAD could correlate with a higher probability of malignancy in the examined PI-RADS 3 lesions, notwithstanding the need for biopsy confirmation (Figures 1, 2).

We performed a decision tree analysis using the CHAID method, which identified a PSAD cut-off of 0.12. This model predicted malignancy with an overall accuracy of 76.7%. In patients with a PSAD less than 0.12, malignancy was found in 23.1% of cases, highlighting the importance of incorporating other clinical factors in the decision-making process for biopsy. For those with PSAD greater than or equal to 0.12, 76.5% were correctly identified as at higher risk for malignancy, underscoring the need for a biopsy (Table 1).

**Table 1:** Accuracy of cancer prediction for PI-RADS 3 lesions on non-contrast-enhanced MRI at PSA density threshold of 0.12 PI-RADS, Prostate Imaging Reporting and Data System; MRI, magnetic resonance imaging; PSA, prostate-specific antigen; CHAID, chi-square automatic interaction detection

Observed	Predicted		
	No Cancer	Cancer	Percent Correct
No cancer	10	3	76.9%
Cancer	4	13	76.5%
Overall percentage	46.7%	53.3%	76.7%
Growing method: CHAID			

Our ROC curve analysis produced a slightly different cut-off for PSAD (0.11) compared to the CHAID analysis (0.12). However, both analyses showed similar trends in predicting higher risk for the presence of prostate cancer, providing a supplementary tool for biopsy decision-making.

In summary, our data suggest a strong correlation between PSAD and the risk of malignancy in PI-RADS 3 lesions in the peripheral zone. It reinforces the importance of biopsy in validating these findings and ensuring accurate diagnosis.

## Discussion

Our study sought to investigate the potential of PSAD in defining high-risk PI-RADS 3 lesions located in the peripheral zone using non-contrast-enhanced MRI. Given the current constraints of the PI-RADS v2.1 in its capacity to detect clinically significant prostate cancer, refining the risk stratification approach is crucial [11,12]. In addition, the push for limiting the use of contrast agents in MRI presents an opportunity for new methodologies to arise [13-15]. As such, this study contributes to the ongoing exploration of alternative approaches to improve the diagnostic pathway of prostate cancer.

PI-RADS v2.1 is widely accepted as an efficient tool for risk stratification in prostate cancer, providing vital assistance in clinical decision-making [3]. However, the precise categorization of PI-RADS 3 lesions, denoting indeterminate risk, remains a challenging task. This is further complicated by the clinical heterogeneity within this group, which often leads to considerable inter-observer variability [16].

At the same time, the utility of PSAD as a risk stratification tool has been validated in various studies [4,6]. In particular, PSAD has been shown to be valuable in assessing the risk of prostate cancer in patients with PI-RADS 3 lesions [17]. Notwithstanding, the optimal PSAD cut-off for accurate risk prediction is still under debate.

Given these uncertainties, our study sought to further explore the potential utility of PSAD in stratifying PI-RADS 3 lesions in the peripheral zone on non-contrast-enhanced MRI, while simultaneously addressing the limitations identified in previous studies. The aim was not only to add to the existing evidence base but also to contribute to optimizing patient management strategies in prostate cancer.

While the aim of this study was to evaluate the role of PSAD in risk stratification in the presence of PI-RADS 3 lesions, it is essential to note that we did not strictly distinguish between the detection of prostate cancer and clinically significant prostate cancer. This approach was intentional due to the common discrepancy observed in Gleason scores on biopsy and prostatectomy in the same patient, as noted in existing literature [9,10] and our practice, including the current study cohort. While this could be seen as a limitation, we believe it adds a level of realism and practicality to our study given the inherent uncertainties in clinical practice.

Our analysis suggests that an elevated PSAD may be indicative of an increased likelihood of malignancy in PI-RADS 3 lesions located in the peripheral zone. Consistent with previous studies [18,19], these results underscore the potential of PSAD in the risk stratification of prostate cancer, particularly for guiding biopsy decision-making. Our analysis suggested a PSAD cut-off value of 0.12. While this threshold was slightly lower than previously reported values [20], it should be interpreted with caution. Although patients with a PSAD below this threshold demonstrated a lower likelihood of malignancy, this negative result should not definitively exclude the possibility of cancer. In our study, we noticed a slight discrepancy between the PSAD cut-off values generated by the CHAID analysis and the ROC/Youden analysis. Although the ROC and Youden analyses are considered more robust for cut-off determination, we opted for the CHAID analysis because we initially intended to include age as a variable in the decision-making process. In this respect, the CHAID method offers greater flexibility in incorporating multiple variables. However, we acknowledge the slight difference in cut-off values as a limitation and recommend further studies to investigate this discrepancy.

Of note, the relatively high rate of cancer detection in our PI-RADS 3 cohort could be attributable to the decision to biopsy these patients. The biopsy decisions made by the urologists were based on unspecified criteria, potentially leading to an overrepresentation of cancer cases in our sample. This bias underlines the need for standardized and more refined risk stratification parameters, such as the PSAD cut-off proposed in our study.

An important aspect of our study design to highlight pertains to our exclusion criteria. We deliberately excluded cases with low-quality MRI images, lesions classified higher than PI-RADS 3 on the same examination, and instances of acute inflammation on pathology. These choices were made to ensure the accuracy and reliability of our results. Specifically, the exclusion of low-quality images reduces the likelihood of misinterpretation or diagnostic errors due to poor visualization. Likewise, the exclusion of higher classified lesions ensures that our study remains focused on PI-RADS 3 lesions only, maintaining the consistency of our sample population. Additionally, the exclusion of cases with acute inflammation on pathology was decided upon due to the potential for inflammation to cause an increase in PSA levels, which could inadvertently skew the threshold for PSAD stratification. These criteria, while potentially limiting the generalizability of our findings, were crucial in ensuring the reliability of our results and conclusions.

Despite the study's promising findings, several limitations must be considered. The relatively small sample size from a single institution limits the generalizability of the results. Moreover, as an exploratory study, our results need to be validated in larger, prospective studies to ascertain the true clinical value of PSAD in this context. Furthermore, the retrospective nature of the study may introduce selection bias. However, this was mitigated to an extent by the fact that all eligible patients during the study period were included in the analysis.

## Conclusions

Our study underscores the potential utility of PSAD in the identification of higher-risk PI-RADS 3 lesions in the peripheral zone using non-contrast-enhanced MRI. The observed association between increased PSAD and the presence of malignancy suggests that PSAD could be a valuable adjunct in risk stratification for prostate cancer. We propose a PSAD cut-off of 0.12, which could augment biopsy decision-making by aiding in the identification of patients at higher risk who may warrant a biopsy, while potentially reducing unnecessary procedures in lower-risk patients, when aligned with their broader clinical profile. This could, in turn, minimize the need for further contrast-enhanced scans. However, these findings stem from a single-institution study with a small patient sample and, therefore, warrant validation through larger, multi-center studies.
